# AEBP1 is a negative regulator of skeletal muscle cell differentiation in oral squamous cell carcinoma

**DOI:** 10.1038/s41598-024-79061-3

**Published:** 2024-11-09

**Authors:** Fumika Okazaki, Akira Yorozu, Shohei Sekiguchi, Takeshi Niinuma, Reo Maruyama, Hiroshi Kitajima, Eiichiro Yamamoto, Kazuya Ishiguro, Mutsumi Toyota, Yui Hatanaka, Koyo Nishiyama, Kazuhiro Ogi, Masahiro Kai, Kenichi Takano, Shingo Ichimiya, Akihiro Miyazaki, Hiromu Suzuki

**Affiliations:** 1 Department of Molecular Biology, Sapporo Medical University School of Medicine, S1, W17, Chuo-ku, Sapporo, 060-8556 Japan; 2 Department of Oral Surgery, Sapporo Medical University School of Medicine, Sapporo, Japan; 3 Department of Human Immunology, Research Institute for Immunology, Sapporo Medical University School of Medicine, Sapporo, Japan; 4 Department of Otolaryngology-Head and Neck Surgery, Sapporo Medical University School of Medicine, Sapporo, Japan; 5 Project for Cancer Epigenomics, Cancer Institute, Japanese Foundation for Cancer Research, Tokyo, Japan

**Keywords:** AEBP1, ACLP, OSCC, Myoblast, Muscle cell differentiation, Cancer, Cancer microenvironment, Oral cancer, Cell biology, Mechanisms of disease

## Abstract

The tumor microenvironment plays a pivotal role in cancer development. We recently reported that in oral squamous cell carcinoma (OSCC), adipocyte enhancer-binding protein 1 (AEBP1) is abundantly expressed in cancer-associated fibroblasts (CAFs), leading to CAF activation and inhibition of CD8 + T cell infiltration. In the present study, we investigated whether AEBP1 contributes to the destruction and atrophy of muscle tissues in OSCC. By analyzing human skeletal muscle myoblasts (HSMMs), we found that AEBP1 is downregulated during muscle cell differentiation. Transcriptome analysis revealed that AEBP1 knockdown significantly upregulates myogenesis-related genes in HSMMs, and qRT-PCR and western blot analyses confirmed the induction of muscle-related genes, including MYOG, in HSMMs after AEBP1 knockdown. Conversely, ectopic expression of AEBP1 strongly suppressed myogenesis-related genes in HSMMs. Notably, indirect co-culture of HSMMs with OSCC cells led to AEBP1 upregulation and robust suppression of muscle-related genes in HSMMs. Treatment with TGF-β1 also upregulated AEBP1 and suppressed expression of muscle-related genes in HSMMs. Our findings suggest that AEBP1 is a negative regulator of skeletal muscle cell differentiation and that OSCC cells inhibit muscle cell differentiation, at least in part, by inducing AEBP1.

**Supplementary Information:**

The online version contains supplementary material available at 10.1038/s41598-024-79061-3.

## Introduction

In 2022, there were 389,485 new cases of oral cancer, accounting for 2.0% of all cancers, and the number of deaths from this disease reached 188,230, representing 1.9% of all cancer deaths^1^. Approximately 90% of oral cancers are histopathologically classified as squamous cell carcinomas^2^. Among the various sites at which oral cancers occur, the tongue is the most common, comprising 30–40% of all cases. Tumor invasion into skeletal muscle is indicative of a poorer prognosis in cancer^3^. In cases of tongue cancer, muscle invasion leads to a 50% increase in local recurrences, a 93% increase in distant metastasis, and a 55% decrease in disease-free survival at 96 months, compared the disease without muscle invasion^4^.

The tumor microenvironment in oral cancer consists of cancer cells, stromal cells, and the surrounding extracellular matrix (ECM)^5^. We previously showed that adipocyte enhancer-binding protein 1 gene (AEBP1) is a novel cancer stroma-associated gene^6,7^. AEBP1 was initially identified as a transcriptional repressor involved in adipocyte differentiation^8^. One of the transcriptional variants of AEBP1 is also known as aortic carboxypeptidase-like protein (ACLP), a secreted protein that associates with the ECM and is involved in wound healing and fibrosis^9–11^. We previously found that upregulation of AEBP1 in vascular endothelial cells promotes tumor angiogenesis in colorectal cancer^6^. We also reported that AEBP1 is abundantly expressed in cancer-associated fibroblasts (CAFs) in oral squamous cell carcinoma (OSCC)^7^. Upregulation of AEBP1 activates CAFs, and high levels of stromal AEBP1 expression are inversely associated with intratumoral infiltration of CD8 + T lymphocytes^7^. AEBP1/ACLP-induced activation of CAFs has also been reported in pancreatic cancer^12^.

During the above studies, we also observed that AEBP1 is abundantly expressed in stromal cells surrounding the atrophic and damaged muscle structures in tongue OSCC. This finding led us to hypothesize that AEBP1 may be involved in the muscle structure disruption seen in OSCC. In the present study, we show that AEBP1 negatively regulates skeletal muscle cell differentiation and that OSCC cells inhibit differentiation of skeletal muscle cells, at least in part, by inducing upregulation of AEBP1 in muscle cells.

## Results

### Downregulation of AEBP1 during skeletal muscle cell differentiation

**Fig. 1:**
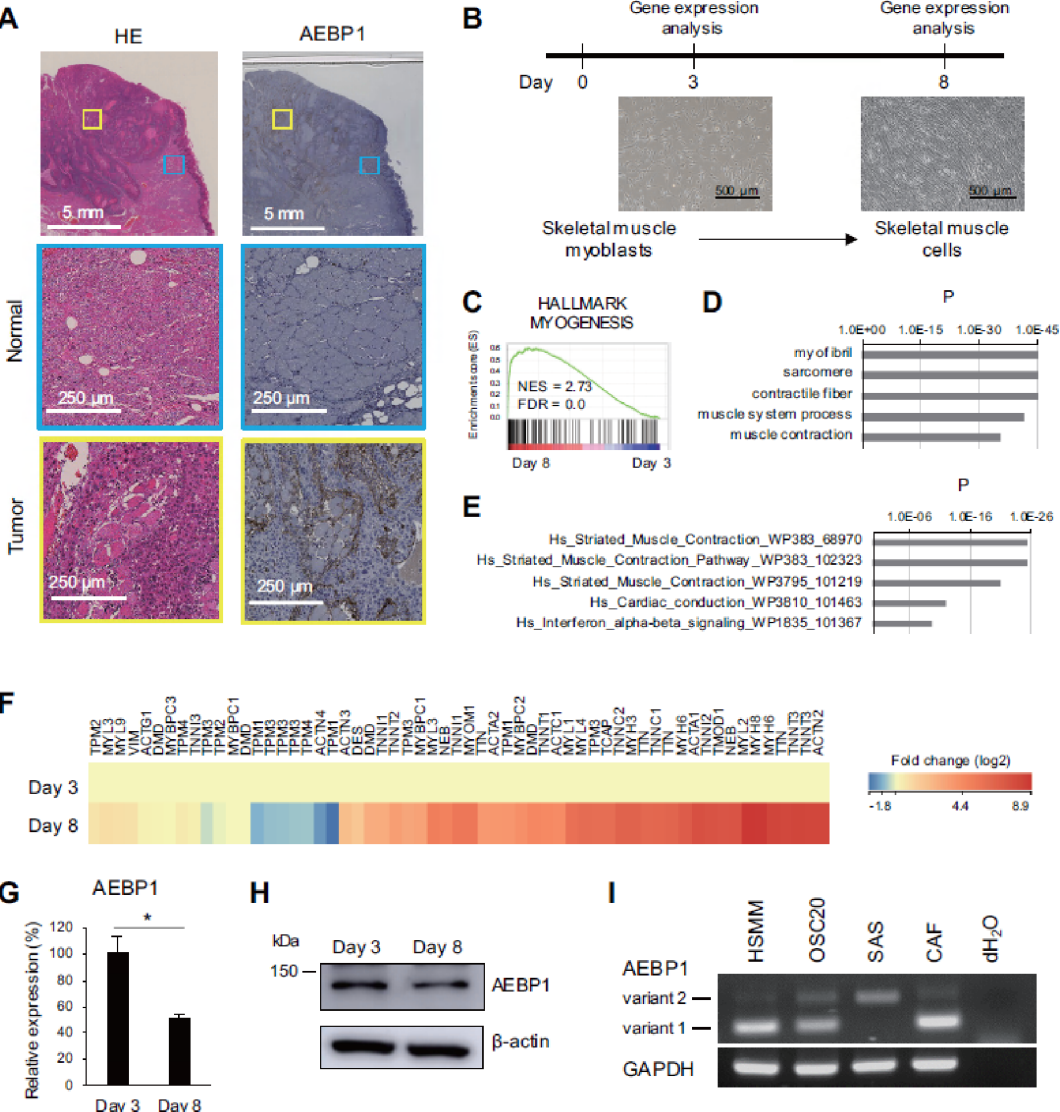
AEBP1 is downregulated during skeletal muscle cell differentiation. (**A**) Immunohistochemical staining for AEBP1 in representative OSCC tissue. Magnified views of the normal and tumor areas indicated by the blue and yellow boxes are shown below. HE, hematoxylin-eosin staining. (**B**) Time course of HSMM culture experiments. Micrographs obtained on days 3 and 8 are shown below. (**C**) GSEA of the hallmark myogenesis gene set using microarray data obtained from HSMMs on days 3 and 8. (**D**) Results of gene ontology (**D**) and pathway (**E**) analyses of the microarray data. (**F**) Heatmap showing expression levels of “striated muscle contraction” genes in (**E**). (**G**) qRT-PCR analysis of AEBP1 in HSMMs at the indicated time points. (*n* = 3). Error bars represent SDs. *P < 0.05. (**H**) Western blot analysis of AEBP1 in HSMMs at the indicated time points. (**I**) RT-PCR of AEBP1 variants in HSMMs. OSCC cell lines (OSC20 and SAS) and CAFs are shown as controls.

In an earlier study, we reported that AEBP1 is abundantly expressed in stromal cells within tongue OSCC tissues. While we showed that a major source of AEBP1 expression is CAFs, we also noted high levels of AEBP1 expression surrounding damaged and atrophic muscle tissues (Fig. 1A). We therefore hypothesized that AEBP1 may be involved in the muscle atrophy and destruction seen during tongue OSCC development. To test that idea experimentally, we utilized commercially available human skeletal muscle myoblasts (HSMMs), which were cultured in standard growth medium for up to 8 days. Total RNA was extracted from the cells on culture days 3 and 8, and gene expression microarray analysis was performed (Fig. 1B). Gene set enrichment analysis (GSEA) showed robust upregulation of myogenesis-related genes and downregulation of cell cycle-related genes on day 8, confirming successful differentiation (Fig. 1C, Supplementary Fig. S1). The microarray analysis identified 3987 probe sets (3301 unique genes) that were upregulated (> 2-fold) in the differentiated cells, and gene ontology and pathway analyses confirmed significant enrichment of muscle-related genes among the upregulated genes (Fig. 1D-F). We then analyzed expression of AEBP1, and found decreased levels of AEBP1 mRNA and protein on day 8 (Fig. 1G, H). These results suggest that AEBP1 is downregulated during skeletal muscle cell differentiation. AEBP1 gene is known to encode two transcription variants, and our RT-PCR results showed that the longer variant, also known as ACLP, is dominantly expressed in HSMMs (Fig. 1I, Supplementary Fig. S2).

## AEBP1 suppresses skeletal muscle cell differentiation

**Fig. 2:**
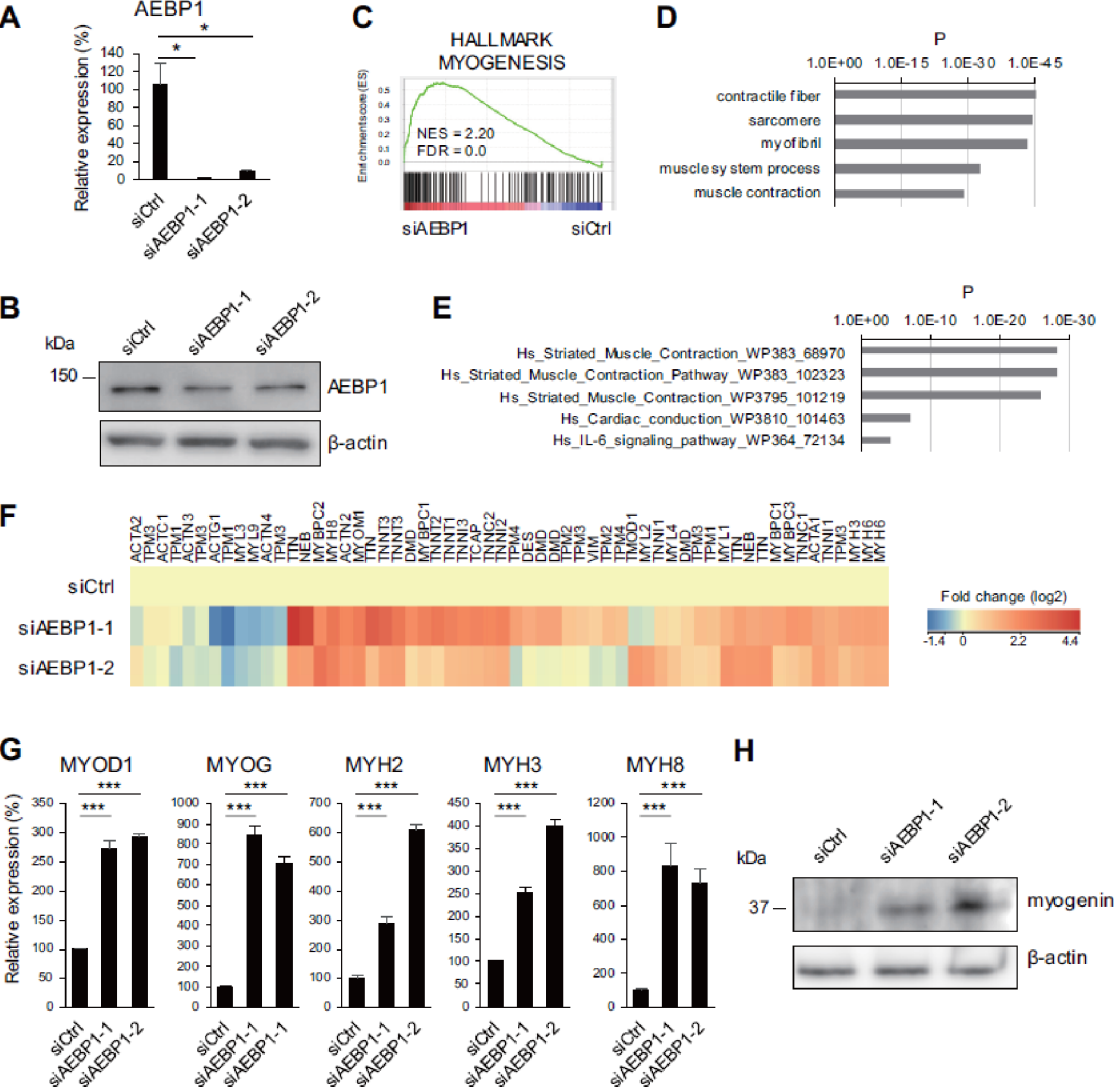
Depletion of AEBP1 promotes skeletal muscle cell differentiation. (**A**) qRT-PCR analysis of AEBP1 in HSMMs transfected with a control siRNA (siCtrl) or siRNAs targeting AEBP1 (siAEBP1-1, siAEBP1-2). (**B**) Western blot analysis of AEBP1 in HSMMs transfected with the indicated siRNAs. (**C**) GSEA of the hallmark myogenesis gene set using microarray data obtained from HSMMs transfected with the control siRNA or siAEBP1-1 and siAEBP1-2. NES, normalized enrichment score; FDR, false discovery rate. (**D**,**E**) Gene ontology (**D**) and pathway (**E**) analyses of genes upregulated after AEBP1 knockdown. (**F**) Heatmap showing expression levels of “striated muscle contraction” genes in (**E**). (**G**) qRT-PCR analysis of the indicated muscle-related genes in HSMMs with indicated siRNAs. (*n* = 3). Error bars represent SDs. ***P < 0.001. (**H**) Western blot analysis of myogenin in HSMMs transfected with the indicated siRNAs.

The results summarized above suggest that downregulation of AEBP1 may be involved in skeletal muscle cell differentiation. To test that hypothesis, we performed knockdown experiments. HSMMs on culture day 3 were transfected with siRNAs targeting AEBP1 (siAEBP1-1 and siAEBP1-2) or with control siRNA and incubated for 72 h. qRT-PCR confirmed the depletion of AEBP1 mRNA in HSMMs transfected with the targeted siRNAs (Fig. 2A), while western blot analysis confirmed the decreased levels of AEBP1 protein (Fig. 2B). We did not observe significant changes in the morphology of HSMMs with AEBP1 knockdown (Supplementary Fig. S3). In contrast, gene expression microarray analysis revealed significant upregulation of myogenesis-related genes in HSMMs transfected with the siRNAs targeting AEBP1 (Fig. 2C). The microarray analysis identified that 465 probe sets (369 unique genes) were recurrently upregulated (> 2-fold) in HSMMs transfected with siAEBP1-1 and those with siAEBP1-2. Gene ontology and pathway analyses showed significant enrichment of muscle-related genes among the genes upregulated after AEBP1 knockdown (Fig. 2D-F). qRT-PCR analysis confirmed the upregulation of muscle-related genes, including myogenic differentiation 1 (MYOD1), myogenin (MYOG) and myosin heavy chain (MYH) genes (Fig. 2G). Increased levels of myogenin expression were also confirmed by western blot analysis (Fig. 2H). The upregulation of muscle-related genes after AEBP1 knockdown was further confirmed in a second lot of commercially available HSMMs (Supplementary Fig. S4).

**Fig. 3:**
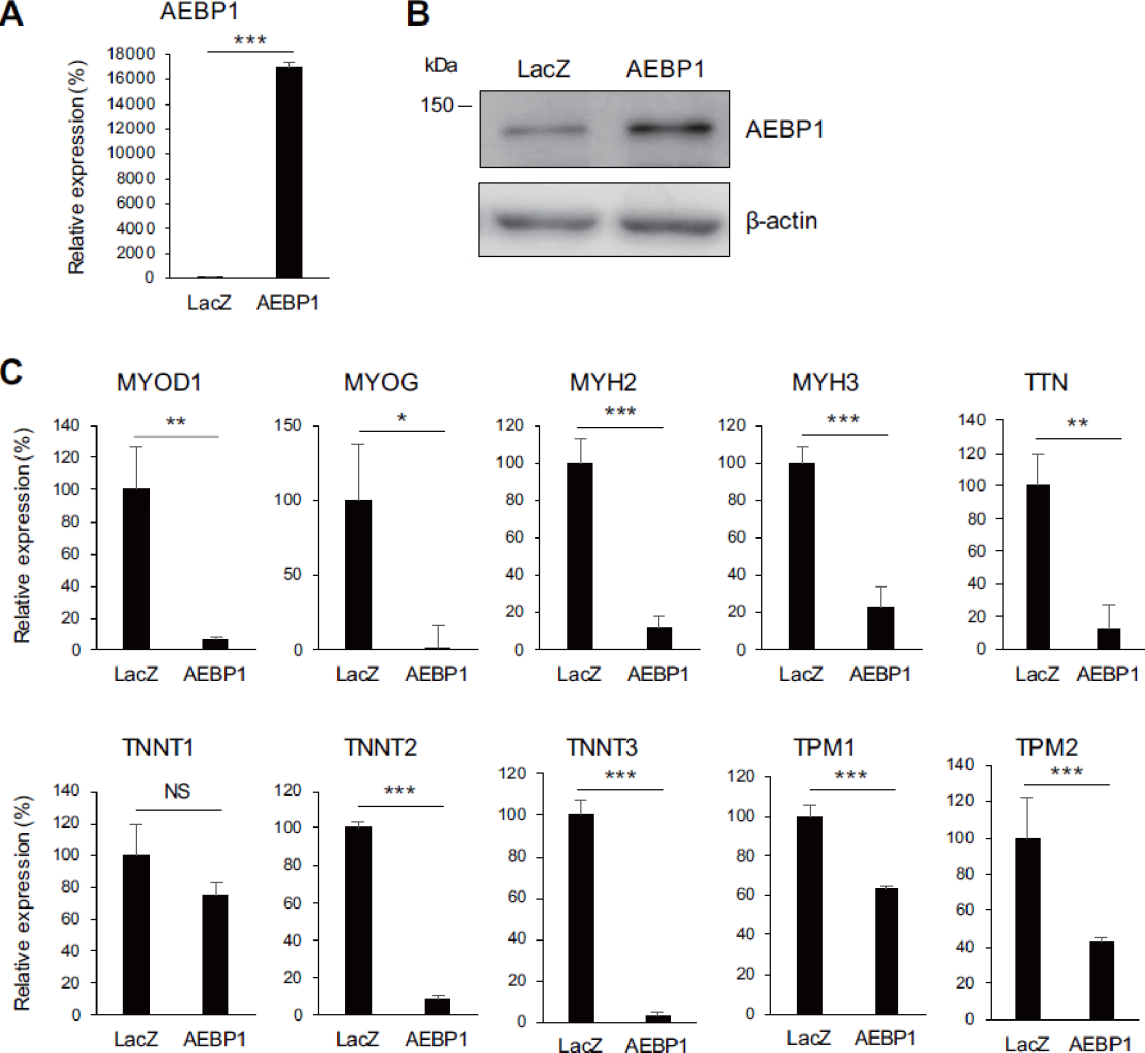
Ectopic expression of AEBP1 suppresses muscle-related genes. (**A**) qRT-PCR analysis of AEBP1 in HSMMs infected with the indicated vectors. (*n* = 3). (**B**) Western blot analysis of AEBP1 in HSMMs infected with indicated vectors. (**C**) qRT-PCR analysis of the indicated muscle-related genes in HSMMs infected with the indicated vectors. (*n* = 3). Error bars represent SDs. **P < 0.01, ***P < 0.001.

To further validate the above results, we also performed overexpression experiments. HSMMs were infected with a lentiviral AEBP1 expression vector or a control vector and cultured for at least 5 days. Ectopic expression of AEBP1 in cells infected with the AEBP1 vector was confirmed by qRT-PCR and western blot analyses (Fig. 3A, B). Notably, we observed decreased expression of muscle-related genes in cells overexpressing AEBP1 (Fig. 3C), and this effect was confirmed in a second lot of HSMMs (Supplementary Fig. S5). These results suggest that AEBP1 inhibits skeletal muscle cell differentiation.

## OSCC cells suppress skeletal muscle cell differentiation

**Fig. 4:**
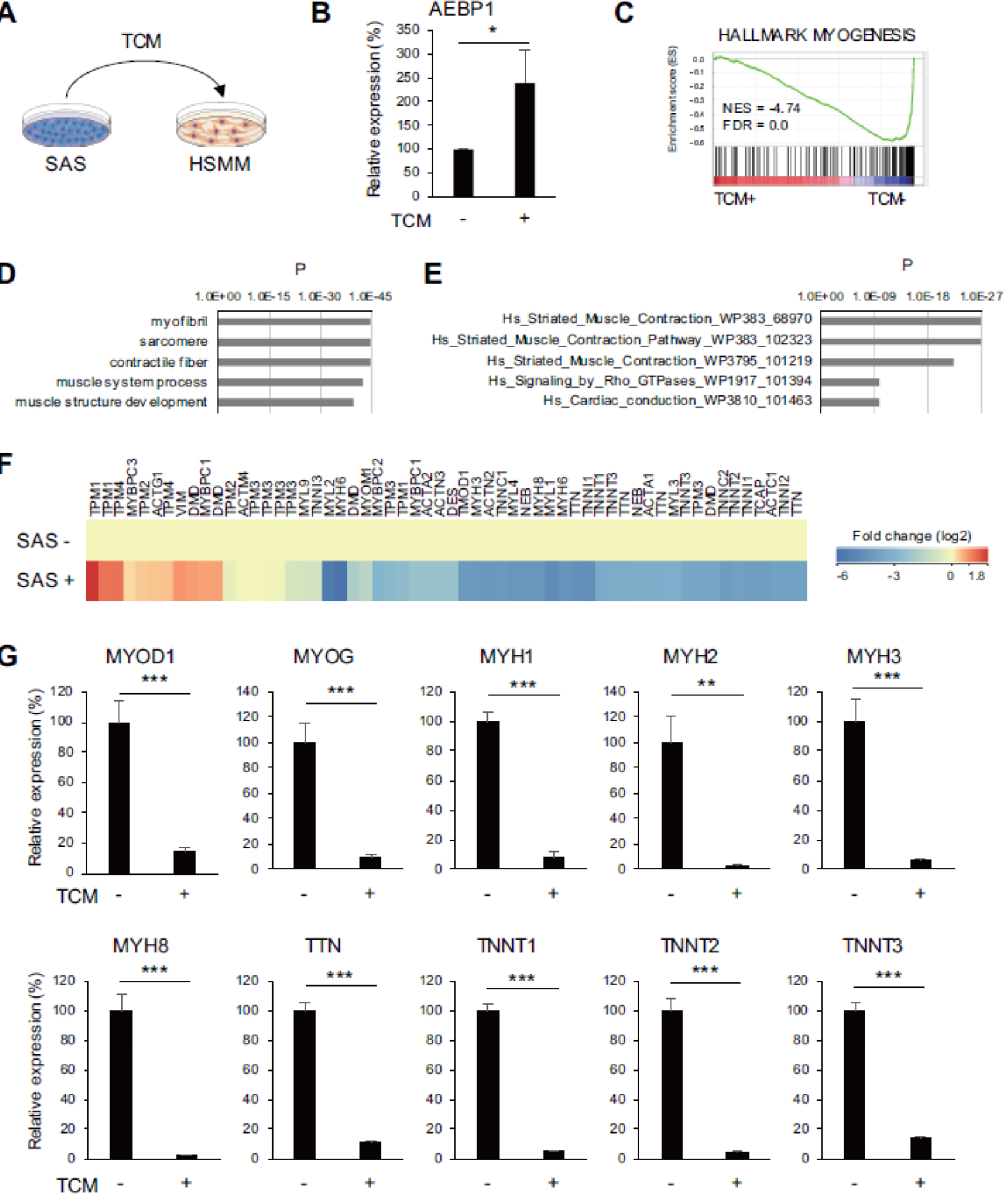
OSCC cells suppress skeletal muscle cell differentiation. (**A**) Schematic representation of the TCM experiments. (**B**) qRT-PCR analysis of AEBP1 in HSMMs cultured with or without TCM. (*n* = 3). (**C**) GSEA of the hallmark myogenesis gene set using microarray data obtained from HSMMs cultured with or without TCM. (**D**,**E**) Gene ontology (**D**) and pathway (**E**) analyses of genes downregulated in HSMMs cultured with TCM. (**F**) Heatmap showing expression levels of “striated muscle contraction” genes in (**E**). (**G**) qRT-PCR analysis of muscle-related genes in HSMMs cultured with or without TCM. Error bars represent SDs. (*n* = 3). *P < 0.05, **P < 0.01, ***P < 0.001.

**Fig. 5:**
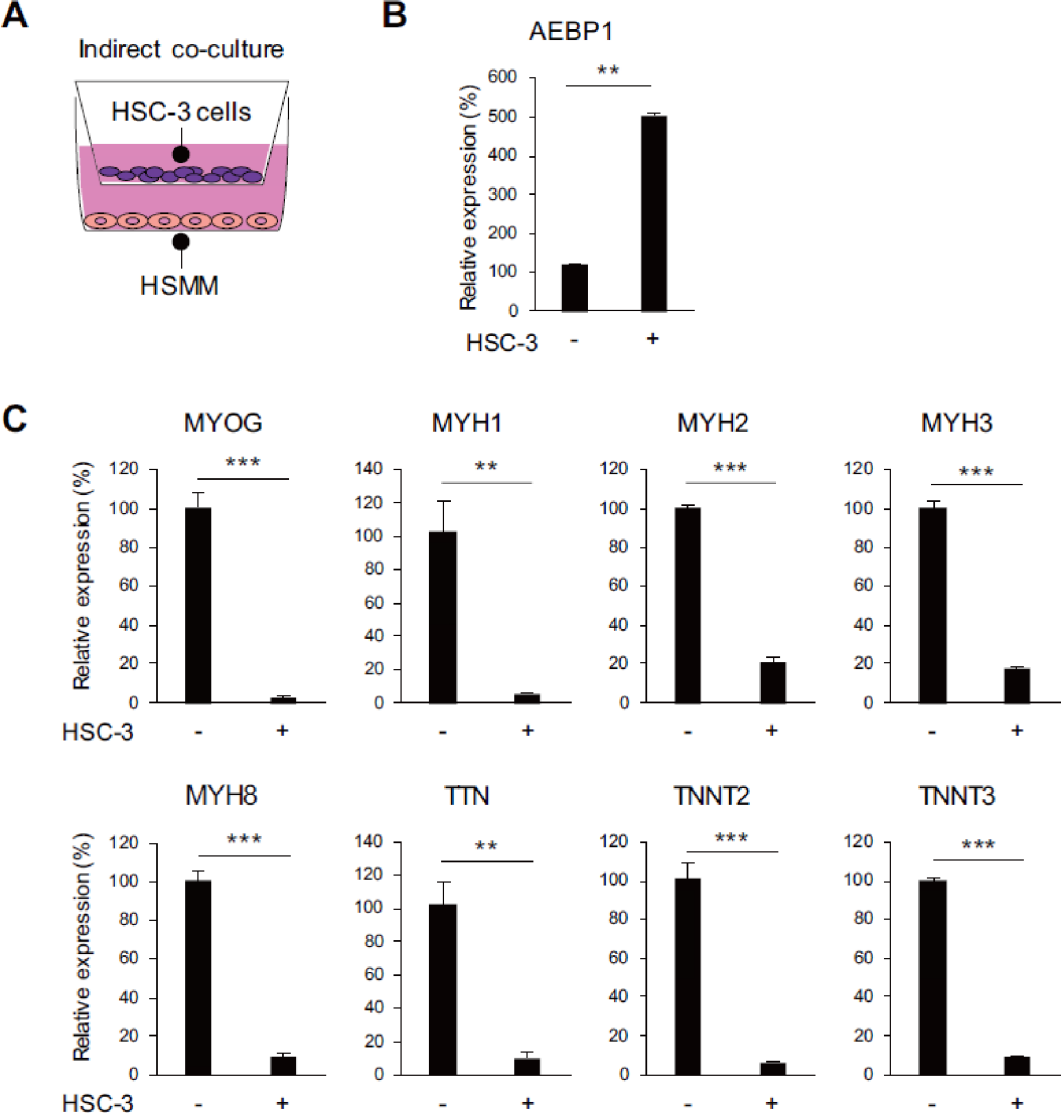
Indirect co-culture experiments using OSCC cells. (**A**) Schematic representation of the co-culture experiments. HSMMs were indirectly co-cultured with HSC-3 cells. (**B**) qRT-PCR analysis of AEBP1 in HSMMs co-cultured with HSC-3 cells and those without co-culture (*n* = 3). (**C**) qRT-PCR analysis of muscle-related genes in HSMMs co-cultured with HSC-3 cells and those without co-culture. Error bars represent SDs. (*n* = 3). *P < 0.05, **P < 0.01, ***P < 0.001.

We next tested whether OSCC cells affect skeletal muscle cell differentiation. When HSMMs were cultured for 72 h in tumor conditioned medium (TCM) prepared with an OSCC cell line (SAS), AEBP1 expression was upregulated (Fig. 4A, B). Gene expression microarray analysis revealed significant suppression of myogenesis-related genes in HSMMs cultured in TCM (Fig. 4C). The microarray analysis identified 3415 probe sets (2618 unique genes) that were downregulated (> 2-fold) in HSMMs cultured in TCM, and gene ontology and pathway analyses showed significant enrichment of muscle-related genes among the downregulated genes (Fig. 4D-F). qRT-PCR analysis confirmed the downregulation of muscle-related genes in HSMMs cultured in TCM (Fig. 4G). Upregulation of AEBP1 and suppression of muscle-related genes were also observed in HSMMs indirectly co-cultured with OSCC cells using transwell plates (Supplementary Fig. S6). Moreover, indirect co-culture with another OSCC cell line (HSC-3) similarly induced upregulation of AEBP1 and downregulation of muscle-related cells in HSMMs (Fig. 5A-C). Finally, we investigated whether OSCC cells also affect differentiated skeletal muscle cells. We found that AEBP1 was upregulated while several muscle-related genes, including MYOD1, MYOG and MYH8, were downregulated in commercially available human skeletal muscle cells (SkMCs) indirectly co-cultured with OSCC cells (Supplementary Fig. S7).

## Transforming growth factor-β1 (TGF-β1) upregulates AEBP1 and inhibits skeletal muscle cell differentiation

**Fig. 6:**
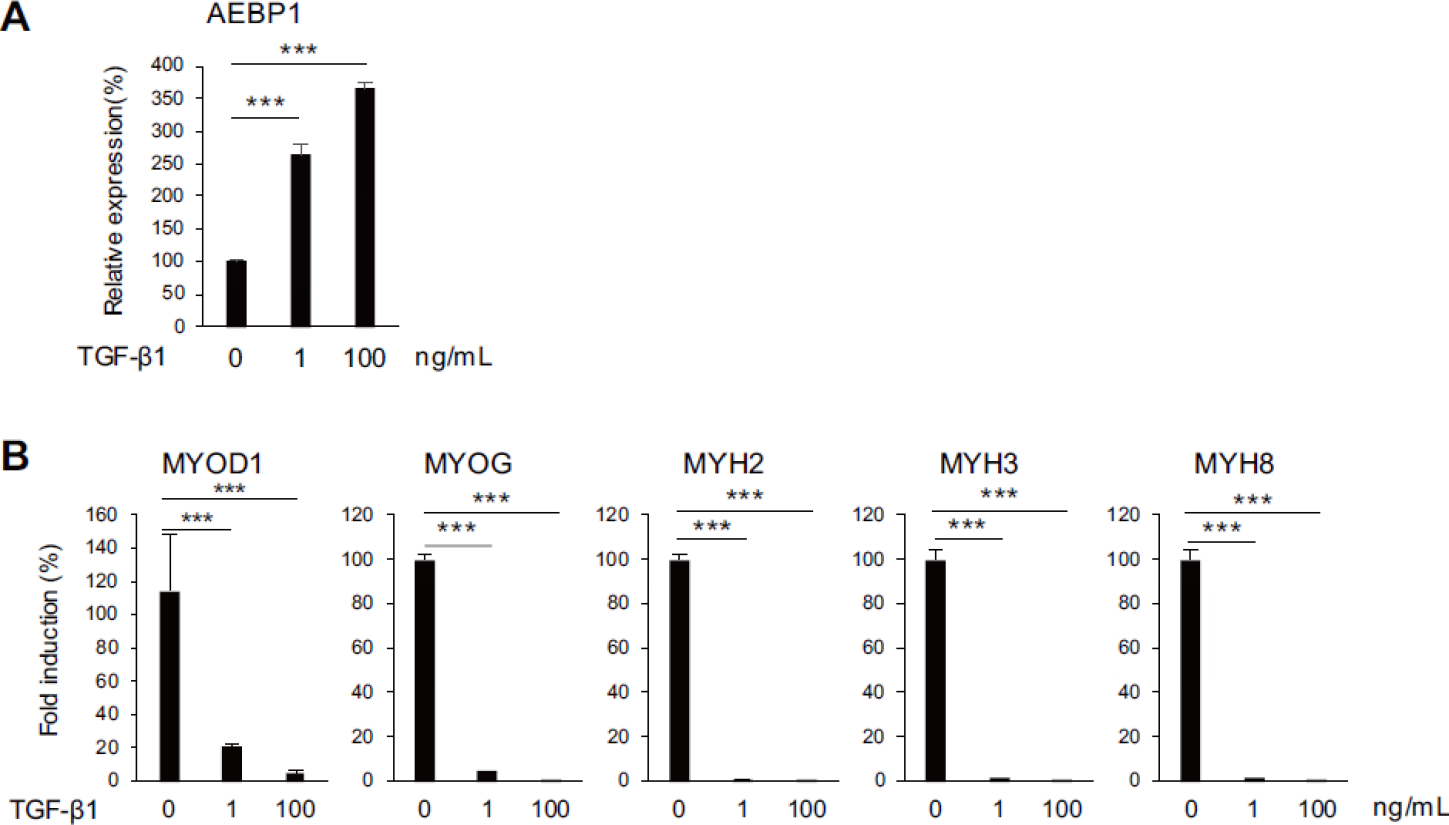
TGF-β1 upregulates AEBP1 and suppresses muscle-related genes in HSMMs. (**A**,**B**) qRT-PCR analysis of AEBP1 (**A**) and muscle-related genes (**B**) in HSMMs treated with the indicated concentrations of TGF-β1. (*n* = 3). Error bars represent SDs. **P < 0.01, ***P < 0.001.

The above results suggest that factors secreted by OSCC cells inhibit skeletal muscle cell differentiation. In earlier studies, we observed that AEBP1 is upregulated in vascular endothelial cells and CAFs indirectly co-cultured with OSCC cells or treated with TGF-β1. In the present study, treating HSMMs with recombinant TGF-β1 induced upregulation of AEBP1 (Fig. 6A) and significant downregulation muscle-related genes (Fig. 6B). These effects were also confirmed in a second lot of HSMMs (Supplementary Fig. S8). Expression of TGFB1 in OSCC cell lines was confirmed using RNA-sequencing (RNA-seq) data (Supplementary Fig. S8). Taken together, our findings suggest that TGF-β1 derived from OSCC cells inhibits skeletal muscle cell differentiation, at least in part, by upregulating expression of AEBP1.

## Secreted AEBP1 suppresses skeletal muscle cell differentiation

**Fig. 7:**
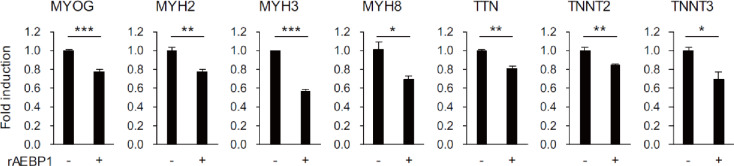
Treatment with recombinant AEBP1 suppresses muscle-related genes in HSMMs. qRT-PCR analysis of muscle-related genes in HSMMs treated with or without recombinant AEBP1 (rAEBP1). Error bars represent SDs. (*n* = 3). *P < 0.05, **P < 0.01, ***P < 0.001.

We next sought to elucidate the mechanism by which AEBP1 inhibits skeletal muscle cell differentiation. AEBP1 was originally identified as a transcriptional repressor in 3T3 preadipocytes. To investigate the interaction between AEBP1 and genomic DNA in HSMMs, we conducted chromatin immunoprecipitation sequencing (ChIP-seq) in HSMMs with ectopic expression of either AEBP1 or LacZ. The number of AEBP1 peaks detected by ChIP-seq analysis (*n* = 177) was similar to that of LacZ peaks (*n* = 157). Visualization of the ChIP-seq results revealed nearly identical patterns between both cell types, indicating that most of the AEBP1 peaks were likely false positives (Supplementary Fig. S9). We subsequently treated HSMMs with recombinant AEBP1 to determine whether secreted AEBP1 exerts an effect. We observed decreased expression of muscle-related genes in cells treated with recombinant AEBP1, indicating that AEBP1 suppresses skeletal muscle cell differentiation, at least in part, through autocrine and/or paracrine mechanisms (Fig. 7).

## Discussion

With this study, we provide evidence that AEBP1 is likely involved in the muscle destruction and atrophy seen in cases of tongue OSCC. Experiments using HSMMs revealed that AEBP1 acts as a repressor of skeletal muscle cell differentiation and that it is downregulated during differentiation of skeletal muscle cells. We also demonstrated that OSCC cells significantly suppress skeletal muscle cell differentiation, at least in part, by inducing AEBP1 and that the AEBP1-mediated inhibition of muscle cell differentiation is induced through TGF-β1. A previous study demonstrated that AEBP1 is regulated by TGF-β through a mechanism requiring p42/44 MAPK activity in 3T3-L1 preadipocytes, suggesting that a similar mechanism may be involved in the induction of AEBP1 in myoblasts^13^. Skeletal muscle regeneration is a highly orchestrated process in which satellite cells (skeletal muscle stem cells) play an indispensable role^14^. Our results suggest that tongue OSCC cells not only disrupt muscle tissues through direct invasion but also may promote muscle atrophy by suppressing muscle regeneration.

AEBP1 was initially identified through the cloning of cDNAs encoding a transcriptional repressor in 3T3 preadipocytes^8^. AEBP1 plays a crucial role in adipogenesis by repressing the aP2 gene, which is essential for lipid metabolism. AEBP1 expression decreases significantly during adipocyte differentiation, indicating it functions as a negative regulator in preadipocytes. In addition to its role in adipogenesis, AEBP1 has been implicated in metabolic and inflammatory processes^15,16^. For instance, AEBP1 influences macrophage cholesterol homeostasis and inflammation by suppressing key regulators like PPARγ1 and LXRα^15^. This suppression promotes foam cell formation and increases expression of proinflammatory mediators, thereby contributing to atherogenesis^15^.

Taken together with the reports summarized above, our results in the present study indicate that AEBP1 may act as a negative regulator of skeletal muscle cell differentiation by transcriptionally repressing myogenesis-related genes. However, our RT-PCR and western blot analyses suggest that the longer variant of AEBP1, also known as ACLP, is dominantly expressed in myoblasts.

Through cloning of cDNAs from human aortic smooth muscle cells, ACLP was first identified as a non-nuclear AEBP1 isoform containing a 380-amino acid N terminal extension^9,17^. It was subsequently shown to be a secreted protein that associates with the ECM and to play a crucial role in vascular smooth muscle cell differentiation and ECM interactions^9,10^. ACLP contains a discoidin-like domain and a carboxypeptidase-like domain, but the absence of the catalytic residues in the latter suggests it may function more as a binding protein. ACLP is abundantly expressed in collagen-rich tissues, such as the vasculature, dermis, and developing skeleton, and it is essential for embryonic development and wound healing^9–11^. Mice lacking ACLP exhibit impaired abdominal wall development and deficient wound healing, indicating its critical role in those processes^10^. Recent studies have demonstrated that mutations in the AEBP1 gene and resultant alterations in ACLP are linked to a novel variant of Ehlers-Danlos syndrome (EDS)^18,19^. Bi-allelic loss-of-function mutations in AEBP1 cause defective collagen assembly, leading to connective tissue abnormalities characteristic of EDS^18,20^.

Recently, both our group and others have reported increased expression of AEBP1/ACLP in CAFs^7,12^. Additionally, our experiments with recombinant AEBP1 demonstrated that AEBP1 may inhibit skeletal muscle cell differentiation through either autocrine or paracrine mechanisms. Collectively, these findings suggest that CAF-derived AEBP1 may also play a role in the suppression of skeletal muscle cell differentiation in OSCC.

Although our results revealed a previously unknown function of AEBP1 in skeletal muscle cells, there are several limitations to this study. For instance, the mechanism by which AEBP1 inhibits muscle cell differentiation is not fully understood. One of the possibilities is that AEBP1 acts as a transcriptional repressor of muscle-related genes, as mentioned above. However, given that AEBP1 expressed in myoblasts appears to encode the secreted protein ACLP, this possibility seems unlikely. Furthermore, our ChIP-seq analysis did not detect any interaction between AEBP1 and genomic DNA in HSMMs, although we cannot entirely rule out the possibility that the ChIP experiment was unsuccessful. One recent study reported that ACLP promotes lung myofibroblast differentiation via both TGF-β-dependent and independent pathways^21^. A similar mechanism may be involved in the inhibition of muscle differentiation by AEBP1, but further research will be needed to elucidate the mechanism by which AEBP1 inhibits skeletal muscle cell differentiation.

In summary, we demonstrated that AEBP1 acts as a repressor of skeletal muscle cell differentiation. Destruction of muscle tissue and inhibition of muscle regeneration by OSCC cells likely contributes to the establishment of a tumor microenvironment that favors cancer cells, potentially promoting cancer progression. Further elucidation of the mechanism by which AEBP1 inhibits skeletal muscle cell differentiation is expected to lead to the development of new cancer therapies that disrupt the tumor microenvironment.

## Materials and methods

### Tissue samples and cell culture

Primary tongue OSCC tissues were collected from Japanese patients who underwent surgical treatment in the Department of Oral Surgery at Sapporo Medical University, as described previously^7^. Human skeletal muscle myoblasts (HSMMs) were purchased from Lonza (Basel, Switzerland) and cultured in SKBM-2 Skeletal Muscle Cell Growth Basal Medium-2 (Lonza) with SKGM-2 Skeletal Muscle Cell Growth Medium-2 SingleQuots Supplements and Growth Factors (Lonza) as described previously^22^. Human skeletal muscle cells (SkMCs) were purchased from Takara Bio (Kusatsu, Japan) and cultured using a skeletal muscle cell growth medium kit (Takara Bio) as described^22^. Human OSCC cell lines (SAS, OSC20 and HSC-3) and CAFs were obtained and cultured as described previously^7^. To prepare TCM, SAS cells were cultured in a 10 cm dish in DMEM (Nacalai tesque, Kyoto, Japan) containing 10% fetal bovine serum (FBS). After replacing this medium with serum-free DMEM, the cells were incubated for an additional 48 h. The TCM was then collected and filtered through a 0.2 μm filter. HSMMs were then cultured in TCM for 72 h. For indirect co-culture experiments, HSMMs (5 × 10^4^ cells/well in 6-well plates) or SkMCs (1 × 10^5^ cells/well in 6-well plates) were indirectly co-cultured with OSCC cells (1 × 10^5^ cells in transwell inserts) for 72 h. Where indicated, HSMMs were treated for 48 h with 1 to 100 ng/mL TGF-β1 (PeproTech, Cranbury, NJ, USA). Where indicated, HSMMs were treated for 72 h with 10 ng/ml recombinant human ACLP (R&D Systems, Minneapolis, MN, USA), with the medium and recombinant protein replaced every 24 h. This study was approved by the Institutional Review Board at Sapporo Medical University (No. 322 − 38).

### Immunohistochemistry

Immunohistochemical staining using a mouse anti-human ACLP/AEBP1 mAb (1:100 dilution, LS‑C133036; LSBio) was carried out as described previously^7^.

### Gene expression microarray analysis

Gene expression microarray analysis was performed using the SurePrint G3 Human GE microarray v2 (G4851; Agilent Technologies, Santa Clara, CA, USA) as described previously^7^. The microarray data were analyzed using GeneSpring GX version 14 (Agilent Technologies) and GSEA (Broad Institute, Cambridge, MA, USA). The Gene Expression Omnibus accession number for the microarray data is GSE268740.

### Reverse transcription-PCR

Total RNA was extracted using an RNeasy Mini Kit (Qiagen, Hilden, Germany). Single-strand cDNA preparation, reverse transcription PCR (RT-PCR) and quantitative RT-PCR (qRT-PCR) were then performed as described previously^7^. β-actin (ACTB) was used as an endogenous control. Primer sequences are listed in Supplementary Table S1.

### Western blot analysis

Extraction of total cell lysates and western blot analysis were performed as described previously^7^. A rabbit anti-AEBP1 mAb (1:750 dilution, ab168355; Abcam, Cambridge, UK), mouse anti-myogenin (F5D) mAb (1:500 dilution, sc-12732 AC; Santa Cruz Biotechnology, Dallas, Texas, USA), mouse anti-V5 tag (6F5) mAb (1:3000 dilution, 011-23591; FUJIFILM Wako Pure Chemical Corporation, Osaka, Japan), and mouse anti-β-actin mAb (1:5000 dilution, clone AC-15; Sigma-Aldrich, Darmstadt, Germany) were used.

### siRNA and expression vector

For AEBP1 knockdown, 5 × 10^4^ cells were transfected with Silencer Select Pre-designed siRNA (100 pmol each; AEBP1 siRNA1, s1145; AEBP1 siRNA2, s1146; Thermo Fisher Scientific) or Silencer Select Negative Control No. 1 siRNA (Thermo Fisher Scientific) using a TransIT-X2 Dynamic Delivery System (Mirus Bio, Madison, WI, USA). Cells were harvested 72 h after transfection. A lentiviral vector encoding full-length AEBP1 was constructed as described previously, after which cells infected with the vector were cultured for at least 5 days until analysis^6^.

### Chromatin immunoprecipitation sequencing

HSMMs were infected with lentiviral vectors encoding full-length AEBP1 or LacZ as described above. Chromatin immunoprecipitation (ChIP) sequencing was performed as described previously^23^ using a rabbit polyclonal anti-V5 tag antibody (ab15828, Abcam). Sequencing data were mapped to human genome hg38 using bowtie2, peaks were called using MACS2, and results were visualized using deepTools^24^ and Integrated Genomic Viewer (IGV) software (Broad Institute, Boston, MA, USA).

### Data analysis

RNA-seq data from OSCC cell lines (GSE256193) were obtained from the Sequence Read Archive (https://www.ncbi.nlm.nih.gov/sra), after which mapping and quantification were performed using the STAR-RSEM pipeline.

### Statistical analysis

Student’s t tests were used to analyze quantitative variables. Values of p less than 0.05 (2-sided) were considered statistically significant. Statistical analyses were performed using GraphPad Prism 5 (GraphPad Software, La Jolla, CA, USA).

## Electronic supplementary material

Below is the link to the electronic supplementary material.

Supplementary Material 1

Supplementary Material 2

Supplementary Material 3

## Data Availability

The Gene Expression Omnibus accession number for the microarray data is GSE268740.
