# Supplementary figures and images for: AEBP1 is a negative regulator of skeletal muscle cell differentiation in oral squamous cell carcinoma
Source: Sci Rep. 2024 Nov 9;14:27425. doi: 10.1038/s41598-024-79061-3 (PMC11550323; doi:10.1038/s41598-024-79061-3)

Figure 1H

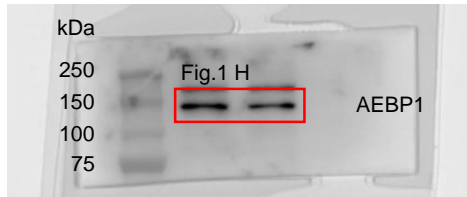

Figures 2B and 3B

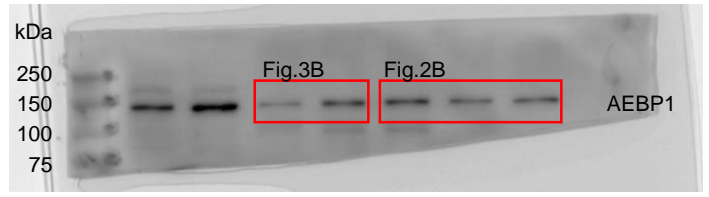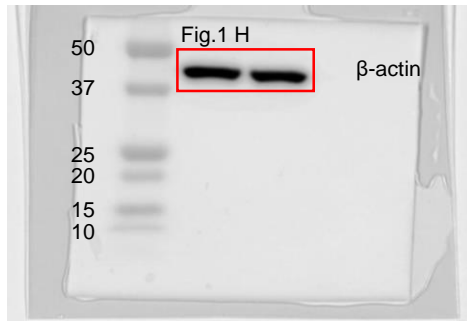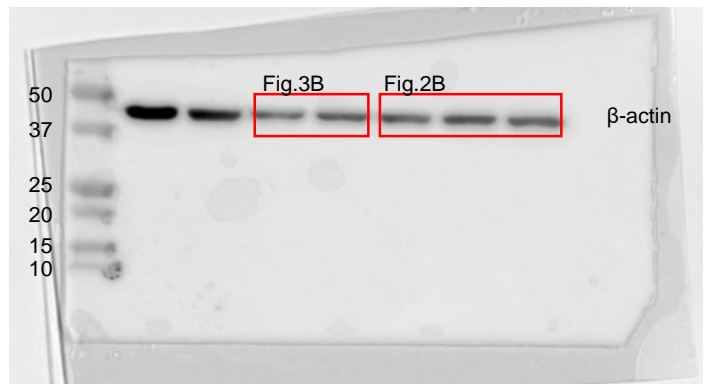

Figure 1I

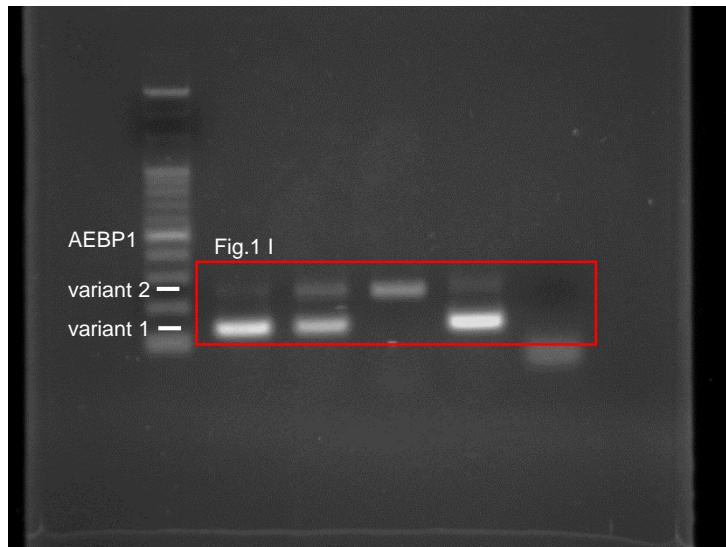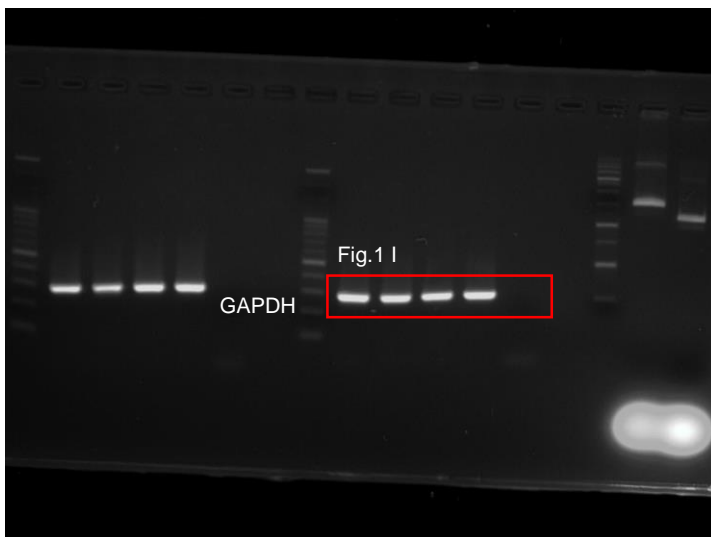

Figure 2H

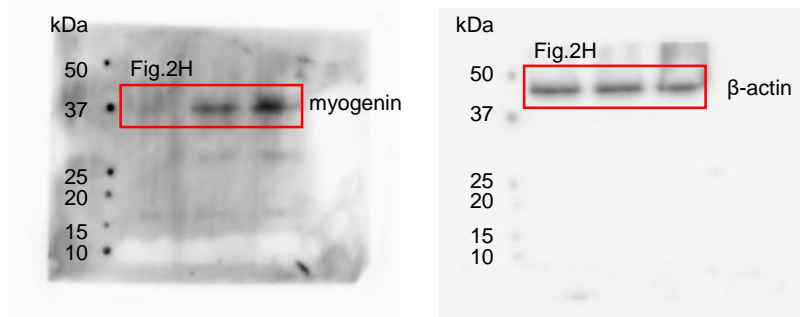

Figure S3B

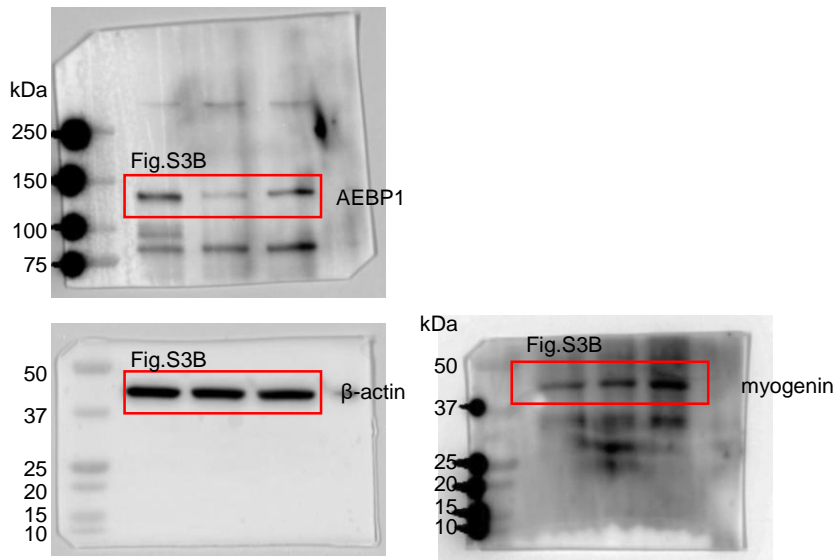

Figure S4B

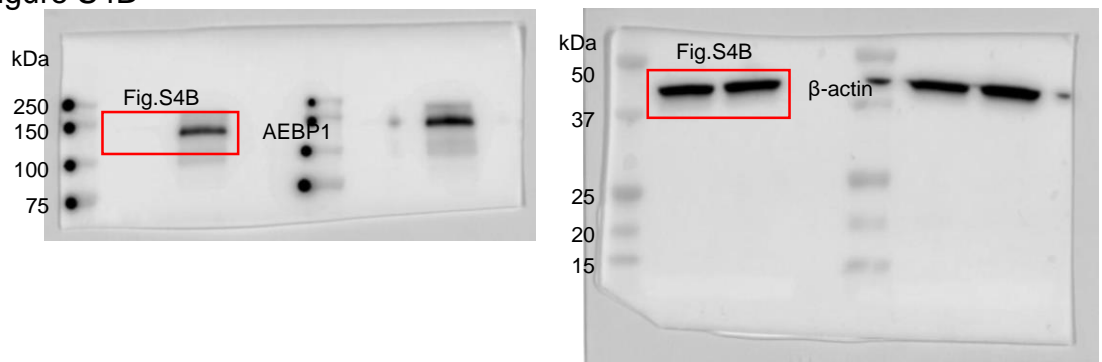

Supplement: Supplementary file 2 — Supplementary Material 2 [file 41598_2024_79061_MOESM2_ESM.pdf]
