# Supplementary material for: AEBP1 is a negative regulator of skeletal muscle cell differentiation in oral squamous cell carcinoma
Source: Sci Rep. 2024 Nov 9;14:27425. doi: 10.1038/s41598-024-79061-3 (PMC11550323; doi:10.1038/s41598-024-79061-3)
Supplement: Supplementary file 3 — Supplementary Material 3 [file 41598_2024_79061_MOESM3_ESM.pdf]

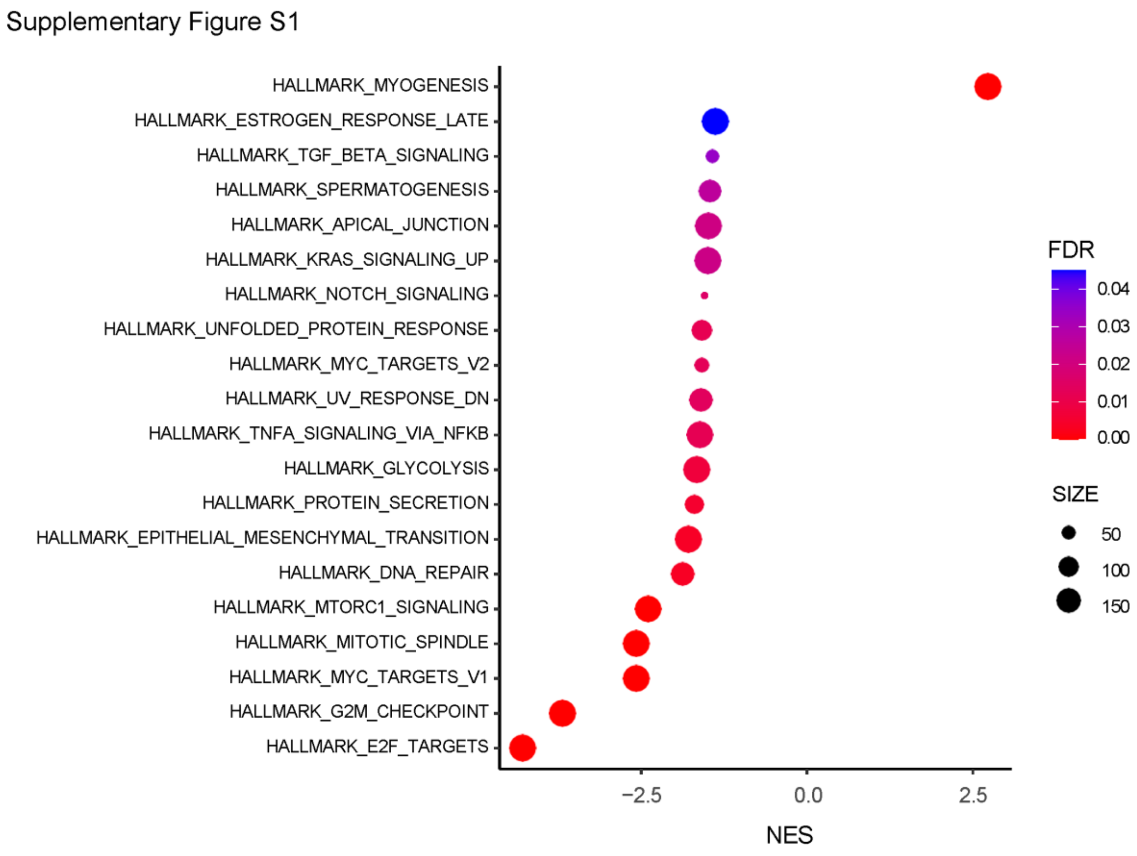

Supplementary Figure S1

GSEA of the hallmark gene sets using microarray data obtained from HSMMs on days 3 and 8. NES, normalized enrichment score; FDR, false discovery rate.

Supplementary Figure S2

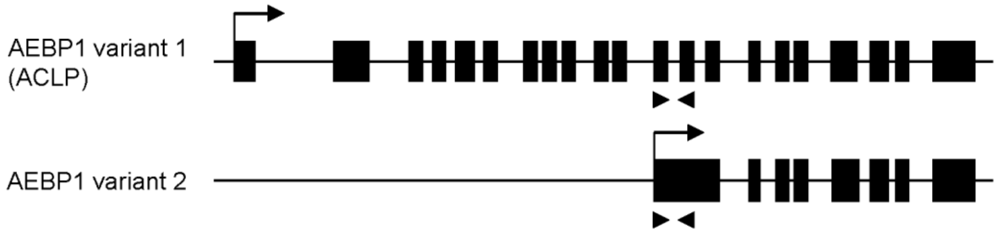

Supplementary Figure S2

Structures of the AEBP1 gene. Locations of RT-PCR primers used to detect transcriptional variants are shown below.

Supplementary Figure S3

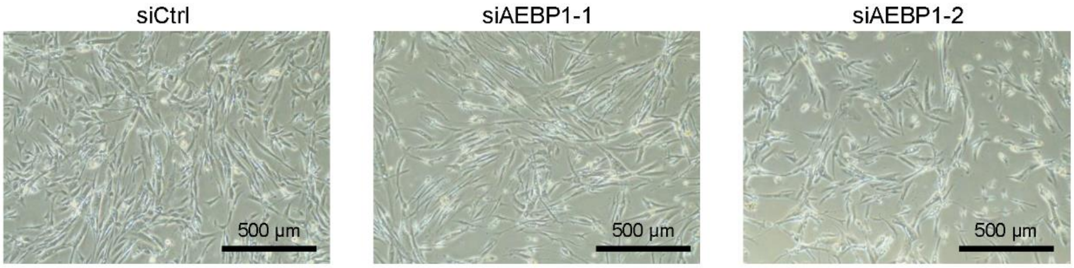

Supplementary Figure S3

Microscopic images of human skeletal muscle myoblasts (HSMMs) transfected with a control siRNA (siCtrl) or siRNAs targeting AEBP1 (siAEBP1-1, siAEBP1-2).

Supplementary Figure S4

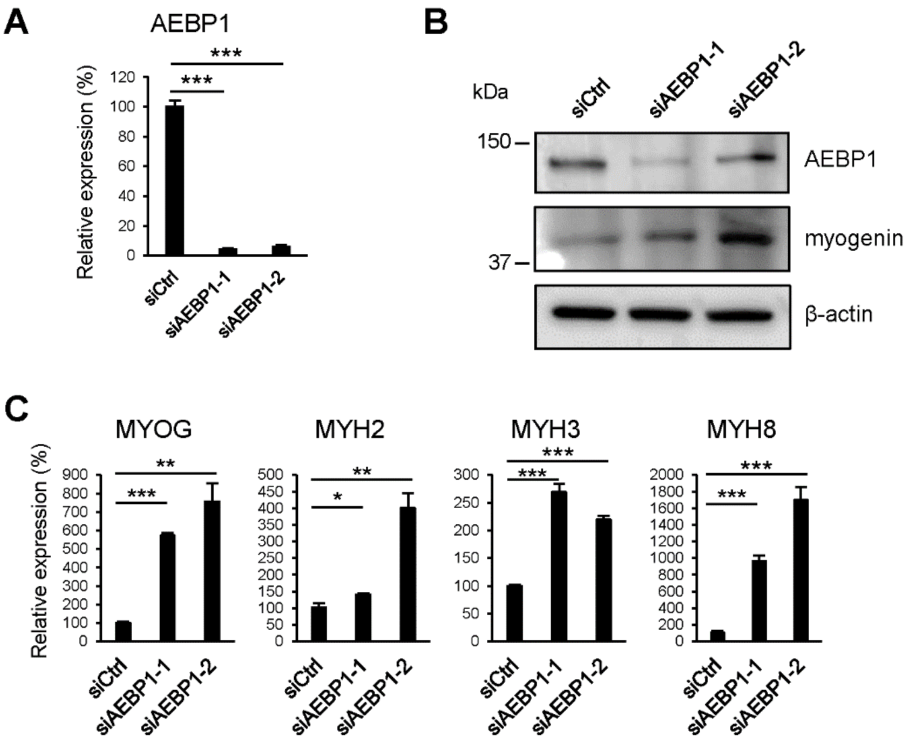

Supplementary Figure S4

AEBP1 knockdown experiments in another lot of HSMMs. (A) qRT-PCR analysis of AEBP1 in HSMMs transfected with a control siRNA (siCtrl) or siRNAs targeting AEBP1 (siAEBP1-1, siAEBP1-2) (n = 3). (B) Western blot analysis of AEBP1 and myogenin in HSMMs transfected with the indicated siRNAs. (C) qRT-PCR analysis of the indicated muscle-related genes in HSMMs transfected with the indicated siRNAs. (n = 3). Error bars represent SDs. *P<0.05, **P<0.01, ***P<0.001.

Supplementary Figure S5

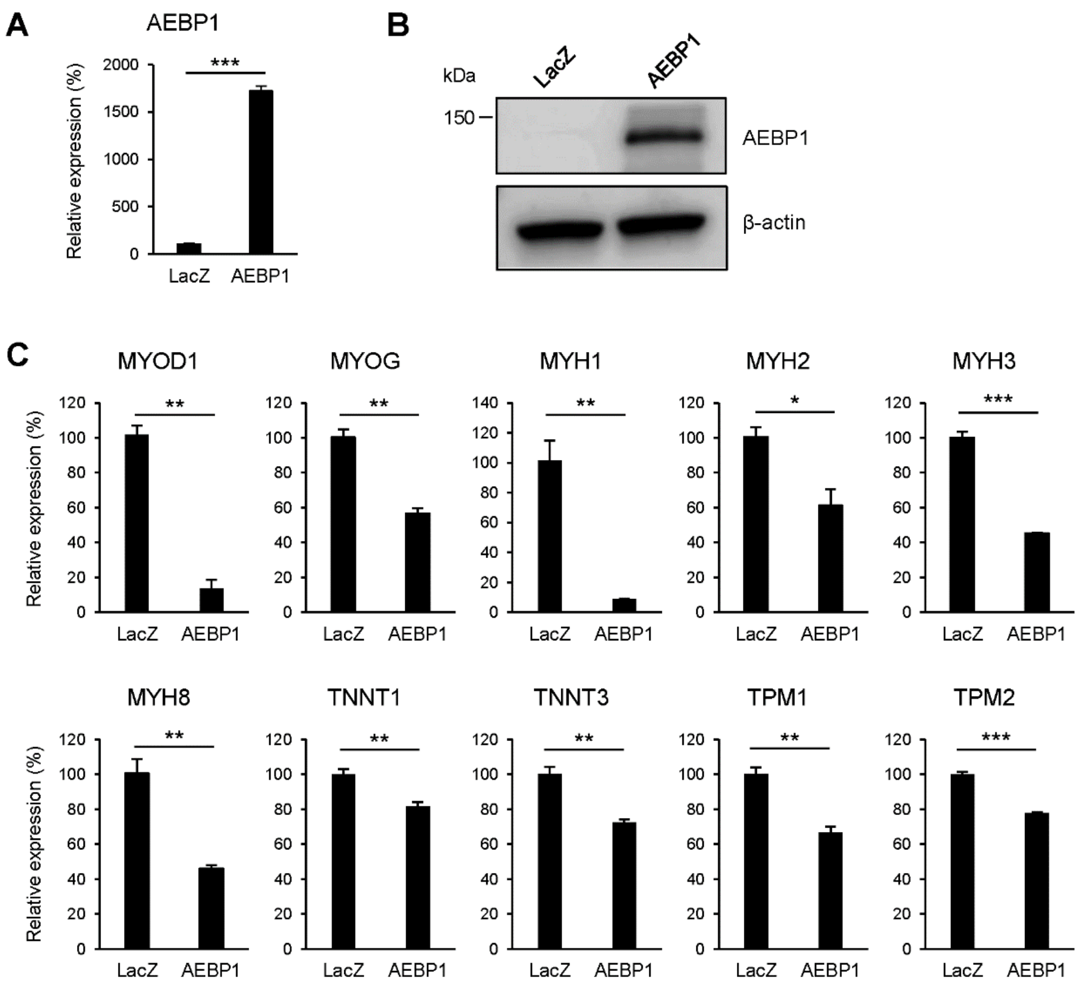

Supplementary Figure S5

AEBP1 overexpression experiments in another lot of HSMMs. (A) qRT-PCR analysis of AEBP1 in HSMMs infected with the indicated vectors. (n = 3). (B) Western blot analysis of AEBP1 in HSMMs infected with the indicated vectors. (C) qRT-PCR analysis of the indicated muscle-related genes in HSMMs with the indicated vectors. (n = 3). Error bars represent SDs. *P<0.05, **P<0.01, ***P<0.001.

Supplementary Figure S6

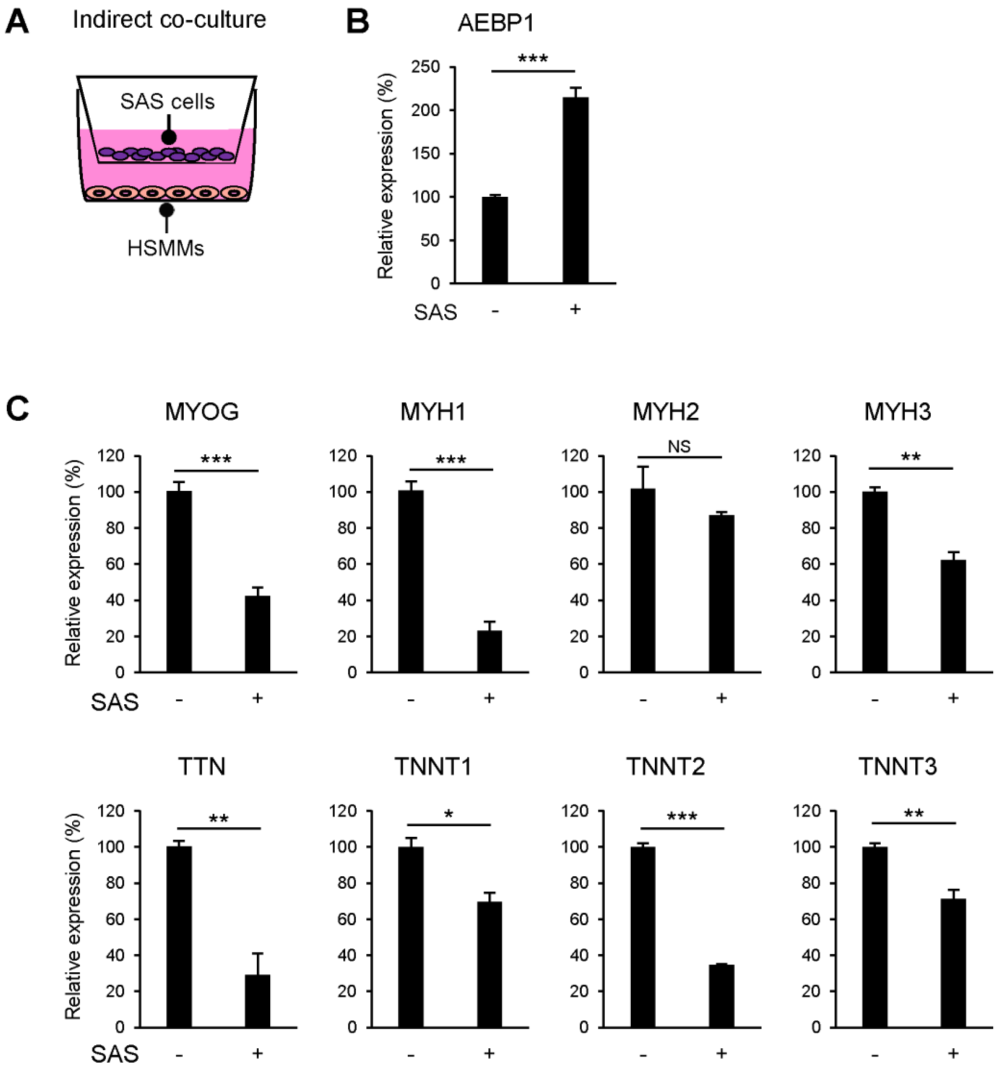

Supplementary Figure S6

Indirect co-culture experiments using HSMMs and OSCC cells. (A) Schematic representation of the co-culture experiments. HSMMs were indirectly co-cultured with SAS cells. (B) qRT-PCR analysis of AEBP1 in HSMMs co-cultured with SAS cells and those without co-culture (n = 3). (C) qRT-PCR analysis of muscle-related genes in HSMMs co-cultured with SAS cells and those without co-culture. Error bars represent SDs. (n = 3). *P<0.05, **P<0.01, ***P<0.001.

Supplementary Figure S7

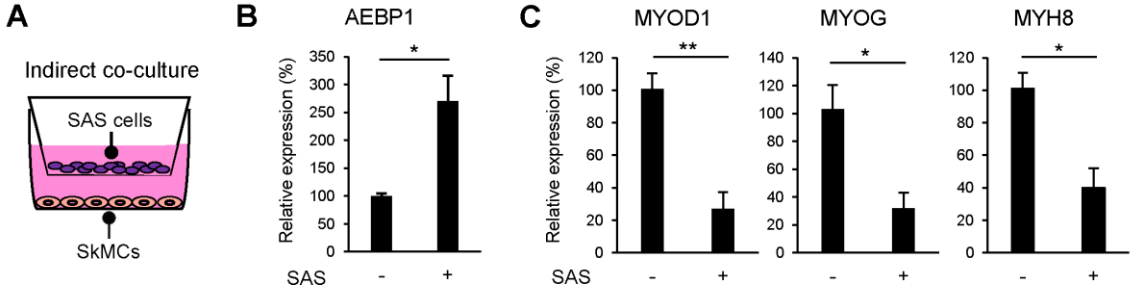

Supplementary Figure S7

OSCC cells suppress muscle-related genes in differentiated skeletal muscle cells. (A) Schematic representation of the co-culture experiments. Human skeletal muscle cells (SkMCs) were indirectly co-cultured with SAS cells. (B, C) qRT-PCR analysis of AEBP1 (B) and muscle-related genes (C) in SkMCs co-cultured with SAS cells and those without co-culture. (n = 3). Error bars represent SDs. *P<0.05, NS, not significant.

Supplementary Figure S8

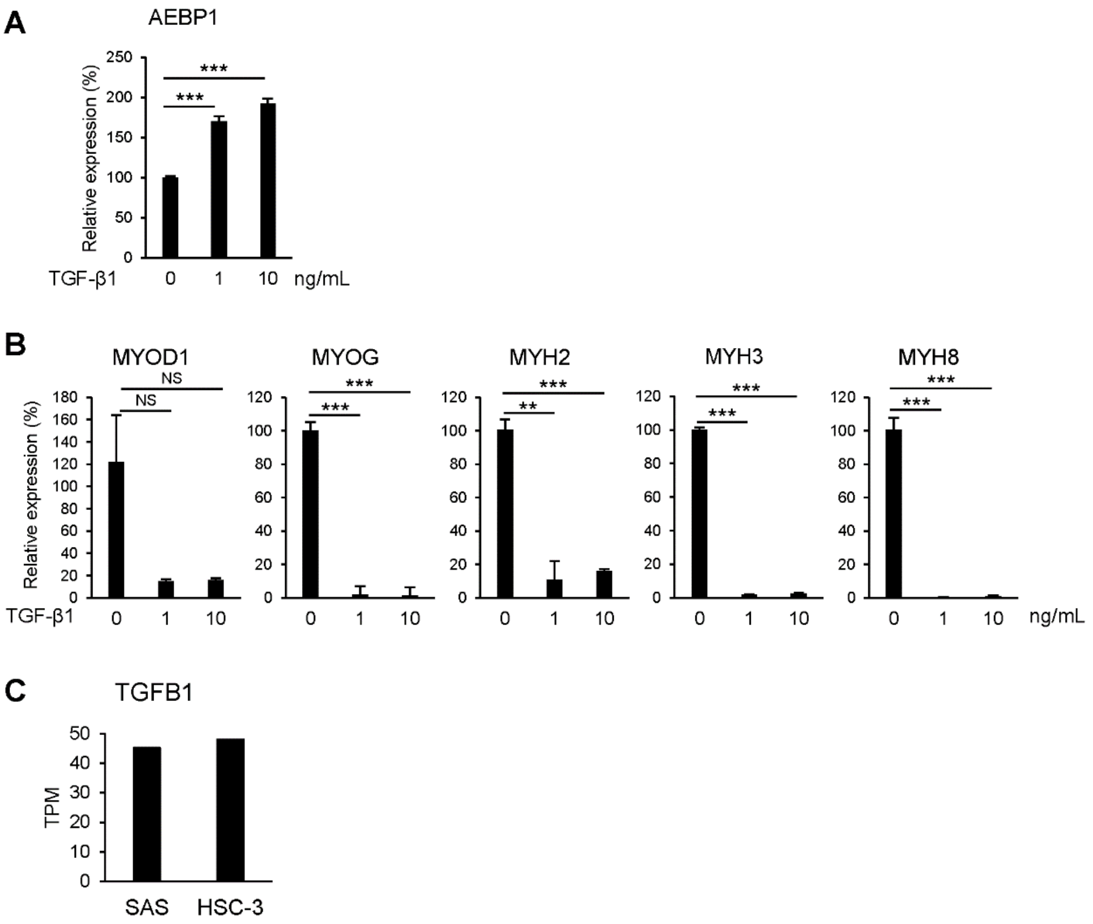

Supplementary Figure S8

Treatment of another lot of HSMMs with TGF-β1. (A, B) qRT-PCR analysis of AEBP1 (A) and muscle-related genes (B) in HSMMs treated with the indicated concentrations of TGF-β1. (n = 3). (C) TGFB1 expression in indicated OSCC cell lines using RNA-seq data (GSE256193). Error bars represent SDs. ****P*<0.001.

Supplementary Figure S9

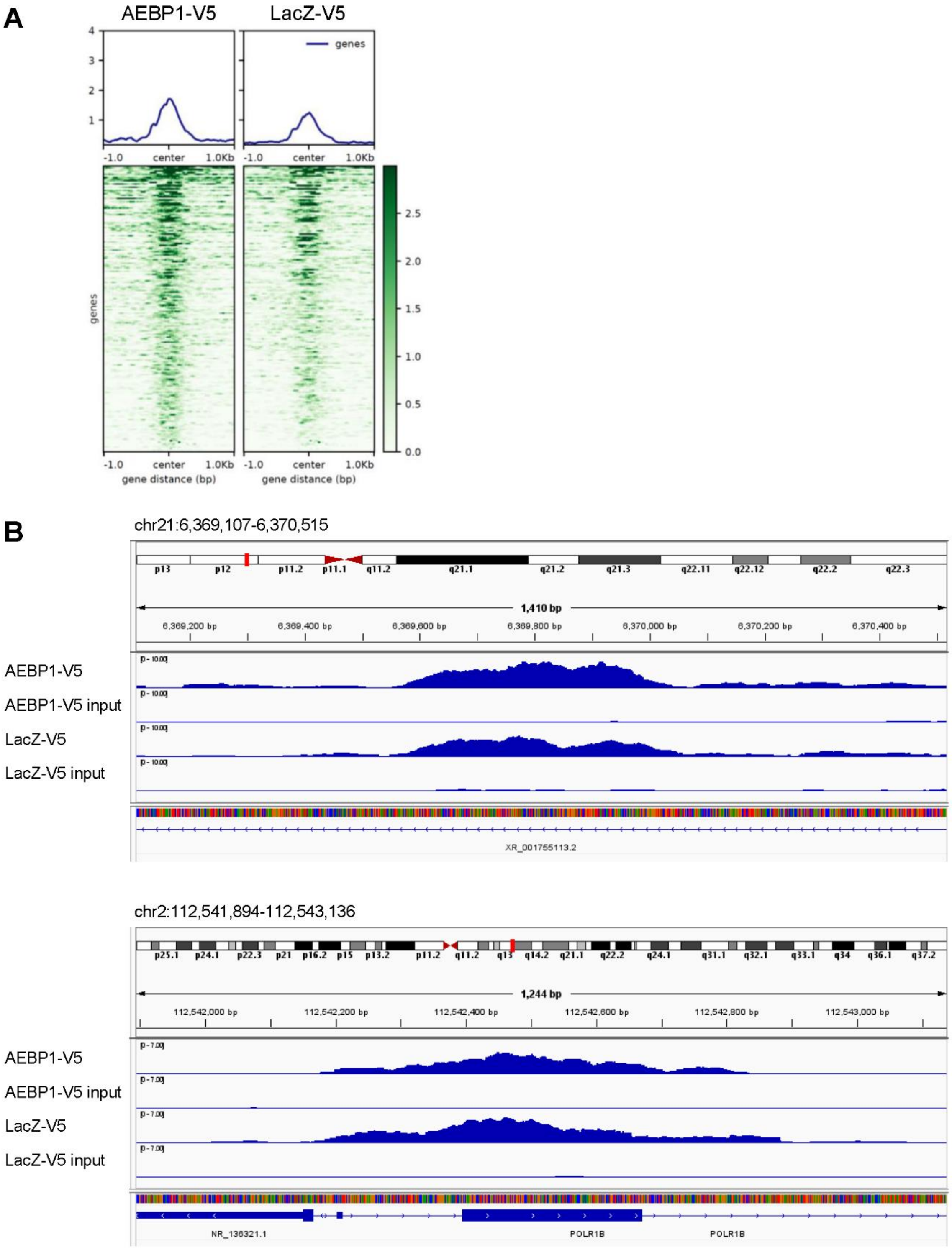

Supplementary Figure S9

Results of chromatin immunoprecipitation-sequencing (ChIP-seq) analysis of ectopically expressed V5-tagged AEBP1 and LacZ in HSMMs. (A) Overview of LacZ-V5 and AEBP1-V5 peaks identified through ChIP-seq. (B) Representative peaks showing the enrichment of LacZ-V5 and AEBP1-V5 at the indicated genomic loci.
